# Using Relaxation Time to characterize biological effects of different mutagens

**DOI:** 10.1038/s41598-020-70600-2

**Published:** 2020-08-18

**Authors:** Xinglin Li, Shuguang Sun, Jingxia Yao, Zhengfeng Sun

**Affiliations:** 1 Tianjin University of Science and Technology, Tianjin, 300457 China; 2 College of Forestry, Nanjing Forestry University, Nanjing, 210037 Jiangsu China

**Keywords:** Agricultural genetics, Plant breeding, Plant evolution, Plant genetics, Plant stress responses, Plant symbiosis

## Abstract

All kinds of mutagenic factors may cause physiological, biochemical and genetic changes of all organisms. To characterize their characteristic biology effects, the concept of Relaxation Time (RT) was introduced for the first time, and the specific process was as follows. After mutation of organisms, the offsprings will be continuingly cultured (or cultivated) to the next generation (Rx). Once a biological effect began to show no significant difference compared to the untreated controls, the Rx was defined as the RT of the effect. In this paper, three kinds of mutagenic factors were selected to treat the seeds or seedlings of *Astragalus sinicus* L., subsequently, the corresponding RT was calibrated. The results showed that the RT was diverse not only among different biological effects but also among different mutagenic factors. For the RT of chemical mutagens and gamma rays, most of which are concentrated on R_1_, whereas the heavy ion beams have significant differences among different tracks. Among biological effects, the SOD activity and superoxide anion free radical content in the Peak region are more prominent, and their RT reaches R_3_ and R_4_, respectively. Thus, the RT may characterize the characteristic biological effects from differently mutagenic factors.

## Introduction

Since X-ray increasing mutation rate was discovered in the last century^1–3^, a series of mutations caused by mutagens have received extensive attention, and it has made many achievements in basic research and applied research^4–6^. And the application of mutagenic means are also constantly innovating in three fields: physical factors from x-rays, ultraviolet rays, gamma rays to various kinds of charged particles^1–3,7–9^, chemical factors from DNA alkylating agents to alkali analogues^10–12^, and biological factors from the introduction of exogenous genes to the knockdown of internal genes^13–15^.

Although all kinds of mutagenic factors can cause biological physiological, biochemical and genetic changes, so far, only the change rate of these biological effect indicators is used to distinguish the differences among these factors or these indicators. However, the change rate of these indexes is influenced by different doses, there is a cross between these ratios. Thus, it is hardly possible to distinguish the characteristic biology effects of different mutagenic factors at present. There are direct and indirect effects in mutagenic biological effects, and direct effects will directly affect indirect effects. As a result, we try to grasp the characteristics of indirect effects to characterize the biological effects of different factors by the Relaxation Time (RT, a concept from thermodynamics) as a new characterization index. The RT indicates the time required for a system to move from an unstable state to a stable state. Since the life system is also a complex and open thermodynamic system^16–18^, when undergoing mutagenesis, the change of the life system is a normal thermodynamic reaction, and when the action of mutagenesis weakens or disappears, the restoration of the initial stable state is also a normal thermodynamic reaction. In the biological system treated by mutagenesis, whether from the molecular expression or from the presentation of the complex traits of the organism, it is more statistically processed parental changes (i.e., the unstable stereotype of the system) from the population than from the offspring of the treatment (i.e., the stable stereotype of the system). Thus, when a mutant was acquired as a mutagenic purpose, only focusing on the individual selection of each breeding link in the offspring, it will completely conceals the process of the effect of the mutagenesis on the life system.

In this study, we selected three kinds of common mutagens, such as gamma-rays, med-high energy heavy ion beams and chemical mutagens, to treat the seeds or seedlings of *Astragalus sinicus* L., respectively. *Astragalus sinicus* L. is a self pollinated legume plant (before flowering, the pollen of every papilionaceous flower has been pollinated). Thus, to save treated time, we selected it as a kind of test materials in this paper. We continuously determined 5 physiological and biochemical indexes of five generations (in 2015–2019) at three developmental stages, and used stable generations (R_x_) as the RT to characterize the characteristic biological effects of mutagens. Those seeds were irradiated using doses of 20, 100, and 200 Gy by gamma rays (i.e., ^60^Co) and 80 meV/u carbon ion beams, respectively; whereas chemical mutagens treated seedlings using three concentrations of alkylating agent diethyl sulfate (DES) and base analogue maleyl hydrazine (MH), respectively.

## Results

### Overview

**Figure 1:**
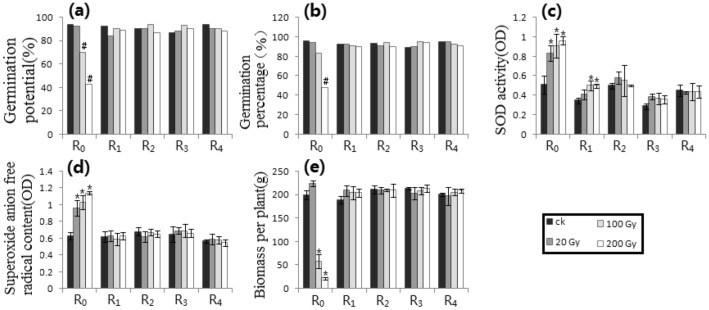
Gamma-ray processing. (**a**) GP_1_. (**b**) GP_2_. (**c**) SODA. (**d**) SAFRC. (**e**) BPP. # shows ≥ 15% difference of mean, compared with respective control. * shows the difference of mean by *p* ≤ 0.05 (Unpaired Sample T-Test), compared with respective control.

**Figure 2:**
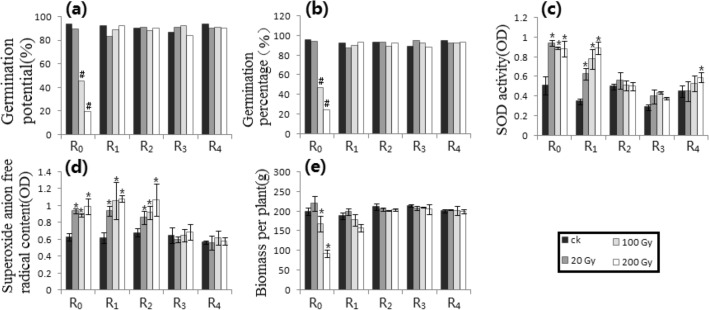
Irradiation processing of Petri dish 1 along the track of heavy ion beams. (**a**) GP_1_. (**b**) GP_2_. (**c**) SODA. (**d**) SAFRC. (**e**) BPP. # shows ≥ 15% difference of mean, compared with respective control. * shows the difference of mean by *p* ≤ 0.05 (Unpaired Sample T-Test), compared with respective control.

**Figure 3:**
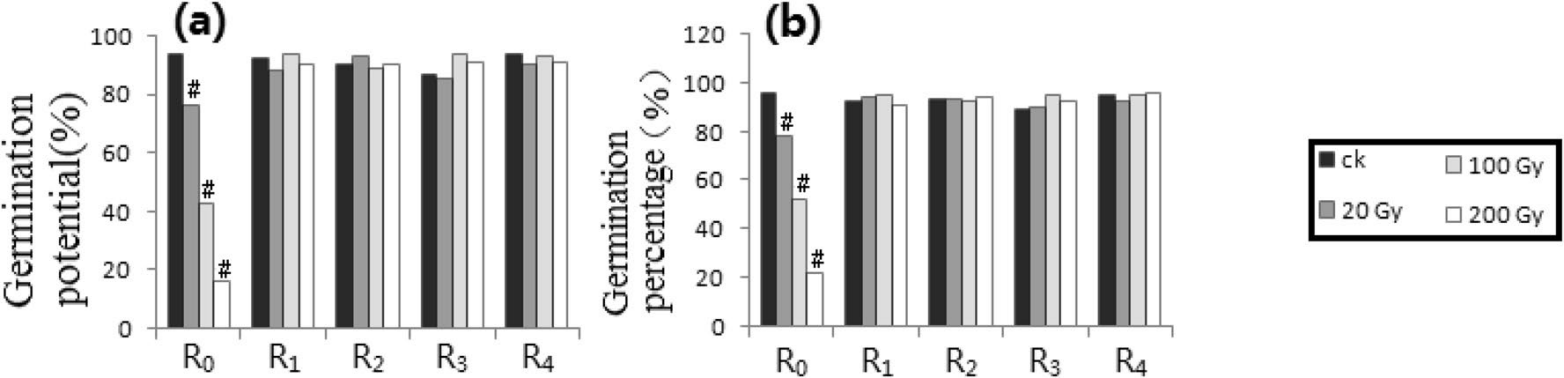
Irradiation processing of Petri dish 2 along the track of heavy ion beams. (**a**) GP_1_. (**b**) GP_2_. # shows ≥ 15% difference of mean, compared with respective control.

**Figure 4:**
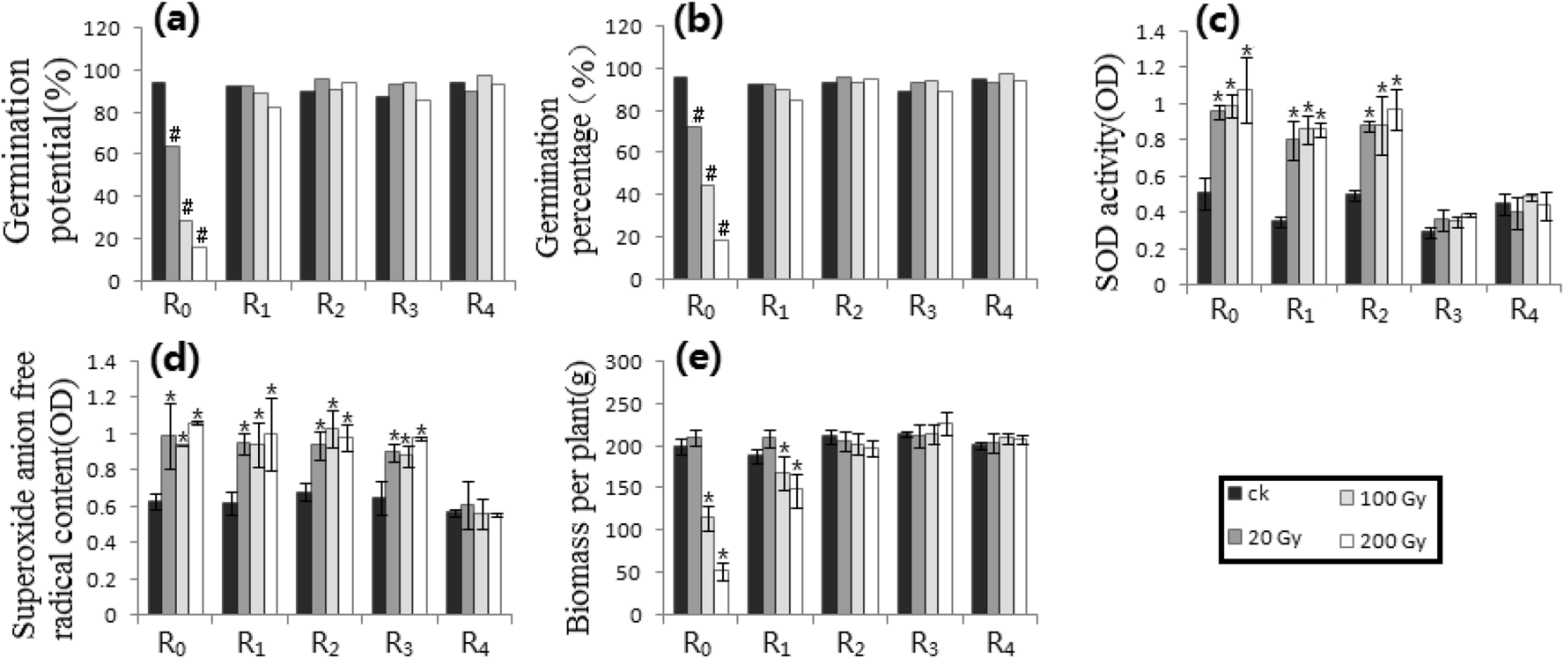
Irradiation processing of Petri dish 3 along the track of heavy ion beams. (**a**) GP_1_. (**b**) GP_2_. (**c**) SODA. (**d**) SAFRC. (**e**) BPP. # shows ≥ 15% difference of mean, compared with respective control. * shows the difference of mean by *p* ≤ 0.05 (Unpaired Sample T-Test), compared with respective control.

**Figure 5:**
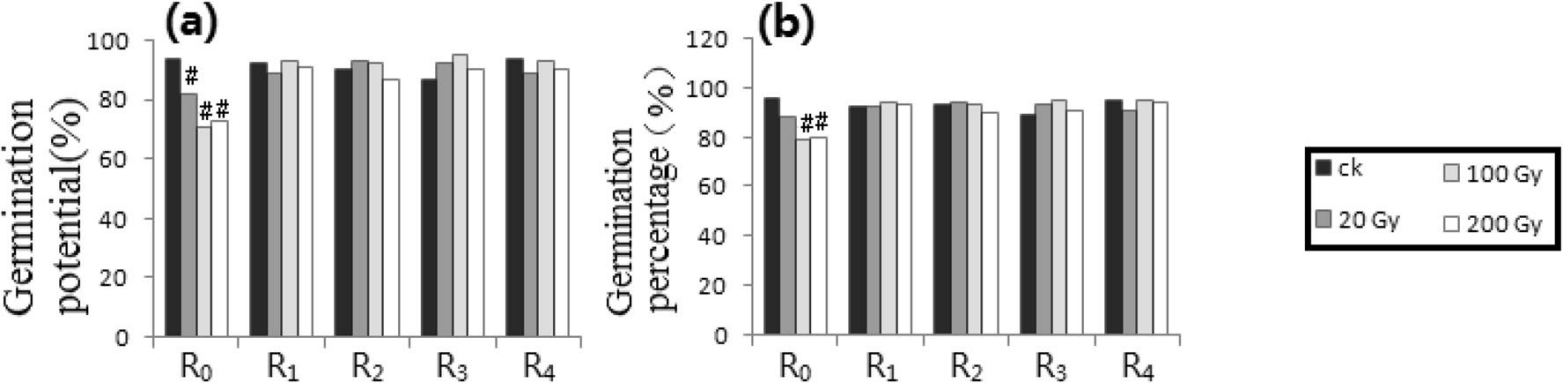
Irradiation processing of Petri dish 4 along the track of heavy ion beams. (**a**) GP_1_. (**b**) GP_2_. # shows ≥ 15% difference of mean, compared with respective control.

**Figure 6:**
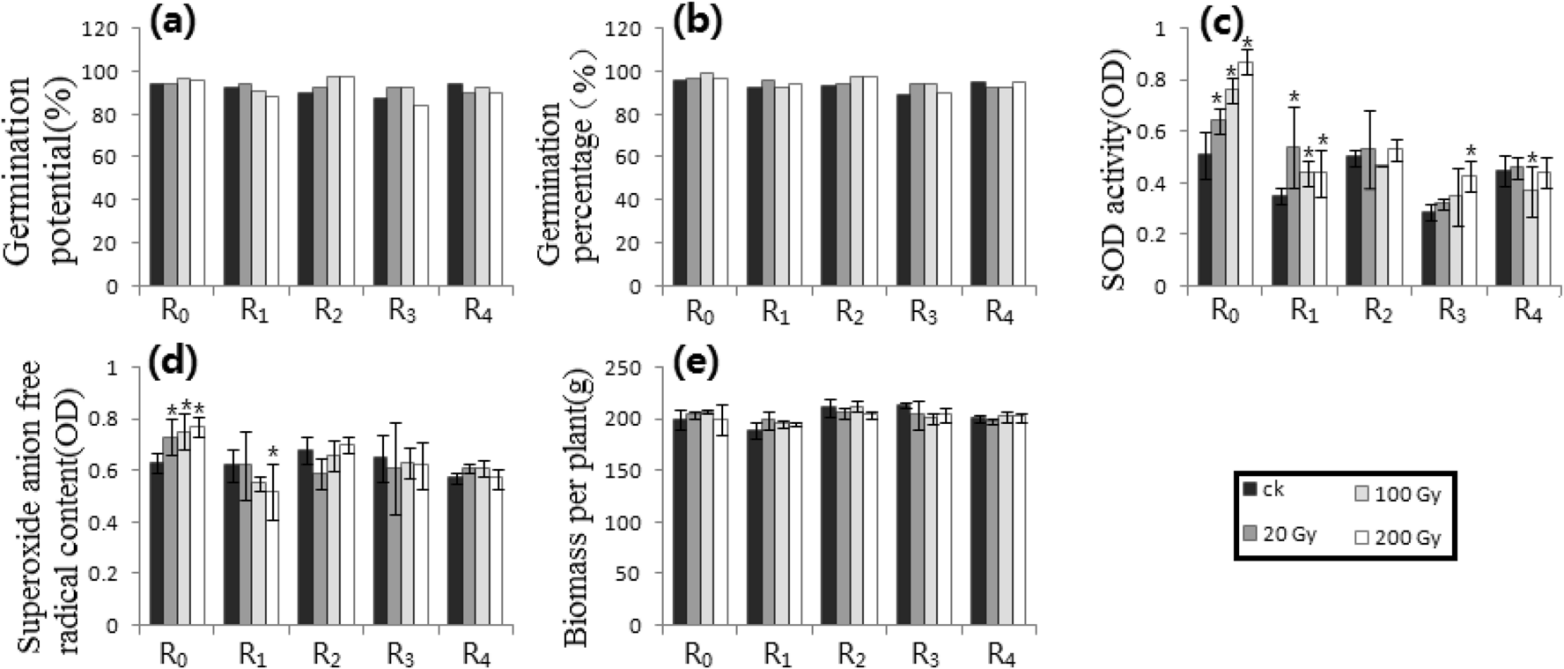
Irradiation processing of Petri dish 5 along the track of heavy ion beams. (**a**) GP_1_. (**b**) GP_2_. (**c**) SODA. (**d**) SAFRC. (**e**) BPP. # shows ≥ 15% difference of mean, compared with respective control. * shows the difference of mean by *p* ≤ 0.05 (Unpaired Sample T-Test), compared with respective control.

**Figure 7:**
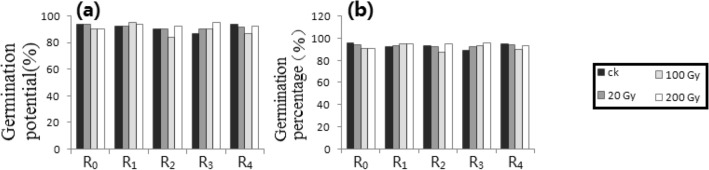
Irradiation processing of Petri dish 6 along the track of heavy ion beams. (**a**) GP_1_. (**b**) GP_2_. # shows ≥ 15% difference of mean, compared with respective control.

**Figure 8:**
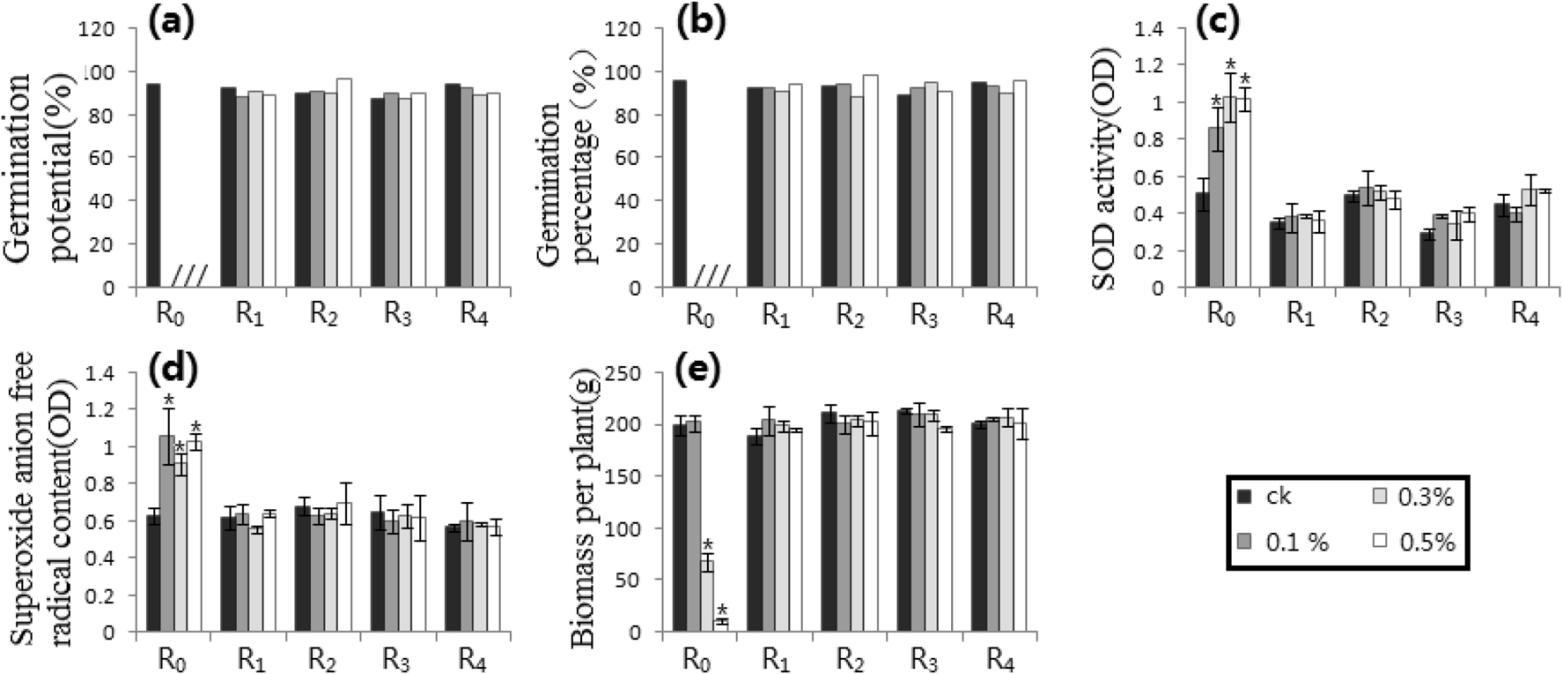
DES processing trials. (**a**) GP_1_. (**b**) GP_2_. (**c**) SODA. (**d**) SAFRC. (**e**) BPP. # shows ≥ 15% difference of mean, compared with respective control. * shows the difference of mean by *p* ≤ 0.05 (Unpaired Sample T-Test), compared with respective control.

**Figure 9:**
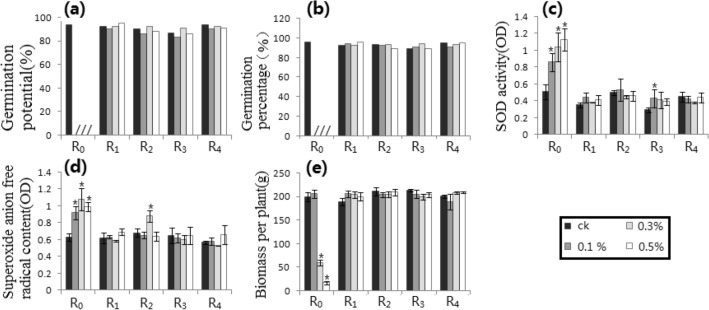
MH processing trials. (**a**) GP_1_. (**b**) GP_2_. (**c**) SODA. (**d**) SAFRC. (**e**) BPP. # shows ≥ 15% difference of mean, compared with respective control. * shows the difference of mean by *p* ≤ 0.05 (Unpaired Sample T-Test), compared with respective control.

All raw data were lined in the Supplementary Materials (Additional information) “S-RD 1”, “S-RD 2”, and “S-RD 3”. Starting from the raw data, compared with the respective control, the difference of germination potential (GP_1_) or germination percentage (GP_2_) was more than 15% and the difference of SOD activity, superoxide anion free radical content (SAFAC) or biomass per plant (BPP) reached a level of *p* ≤ 0.05, which was listed as a significant difference, and the statistical results were shown in Figs. 1, 2, 3, 4, 5, 6, 7, 8 and 9 as follows.

### Gamma-ray trials (Fig. 1)

Compared with the control, only higher doses of the germination potential (GP_1_) and the germination percentage (GP_2_) had more than 15% differences in R_0_ (i.e., RT = R_1_), while other generations (i.e., RT = R_0_) had no significant difference. For the SOD activity, R_0_ (i.e., RT = R_1_) at all doses and R_1_ (i.e., RT = R_2_) at high doses showed significant differences compared with controls (*p* = 0.05). For the superoxide anion free radical content (SAFRC ) at all doses and the biomass per plant (BPP) at high doses, only there was a significant difference in R_0_ (i.e., RT = R_0_) compared with the control, while there was no significant difference in the BPP at low dose (i.e., RT = R_0_). The analysis results were listed in Table 1.

**Table 1:**
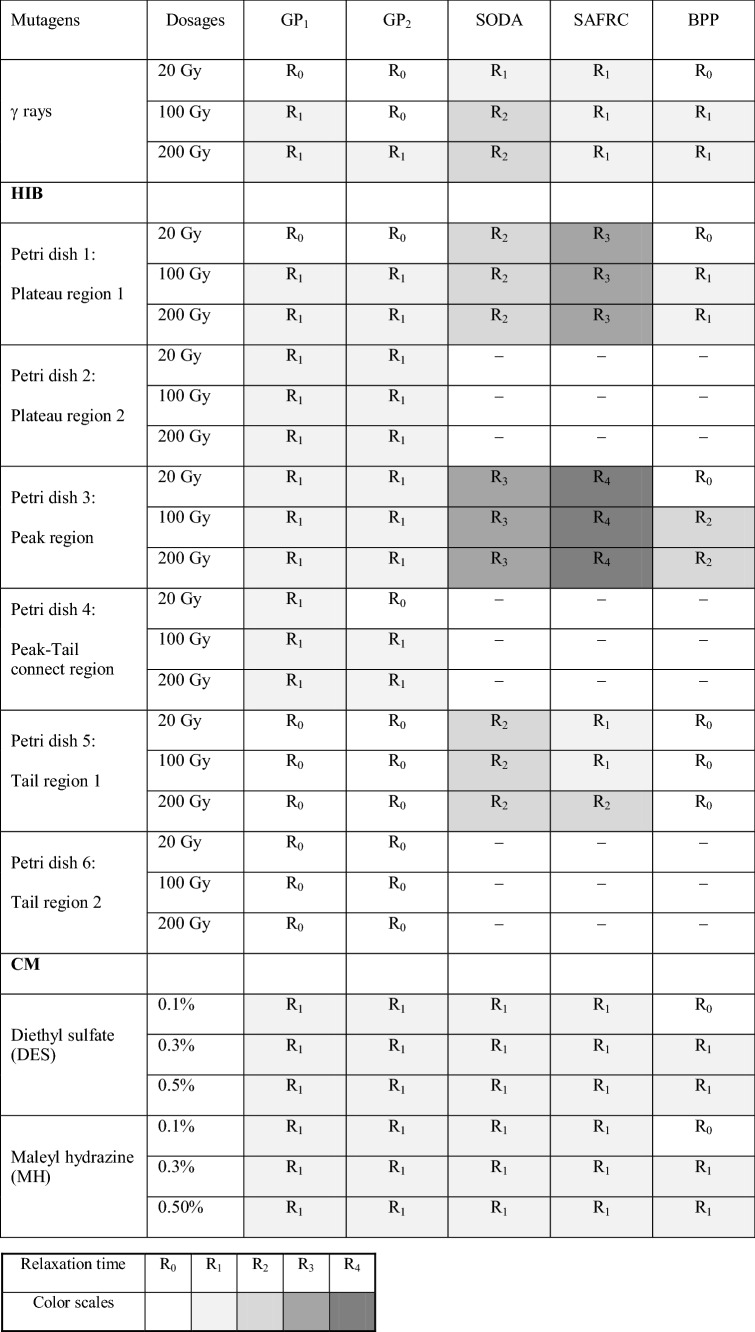
Distribution of the Relaxation Time on physiological and biochemical indexes. HIB, Heavy ion beams. CM, Chemical mutagens. GP_1_, Germination potential. GP_2_, Germination percentage. SODA, SOD activity. SAFRC, Superoxide anion free radical content. BPP, Biomass per plant. “–” shows no determination.

### Heavy ion beam trials (Figs. 2, 3, 4, 5, 6, 7)

Heavy ion beam irradiation is a complex process: six Petri dishes with seeds were stacked along 80 meV/u carbon ion beam tracks and separated into six regions to test after irradiation (see "Methods" for details), respectively. Petri dish 1 and 2, were equivalent to the Plateau regions of the Bragg curve; Petri dish 3 was close to the Peak of the Bragg curve; Petri dish 4 was the Peak-Tail junction region; and Petri dish 5 and 6 were the Tail regions of irradiation.

Figure 2 showed the results of Petri dish 1. Compared with the control, only R_0_ (i.e., RT = R_1_) of the GP_1_ and the GP_2_ had more than 15% differences at higher doses, while other generations (i.e., RT = R_0_) had no significant differences. There were significant differences in R_0_ and R_1_ (i.e., RT = R_2_) in the SOD activity compared to control (*p* ≤ 0.05). There were significant differences in R_0_, R_1_ and R_2_ (i.e., RT = R_3_) of the SAFRC (*p* ≤ 0.05). For the BPP, there was significant difference in R_0_ (i.e., RT = R_1_) at high doses compared with the respective control.

Figure 3 showed the results of Petri dish 2. Only the GP_1_ and the GP_2_ were measured. Compared with the control, only R_0_ generations (i.e., RT = R_1_) of the GP_1_ and the GP_2_ had more than 15% differences.

Figure 4 showed the results of Petri dish 3. For the GP_1_ and the GP_2_, only R_0_ (i.e., RT = R_1_) had more than 15% differences compared with the control. R_0_, R_1_, and R_2_ (i.e., RT = R_3_) of the SOD activity at all doses, R_0_, R_1_, R_2_ and R_3_ (i.e., RT = R_4_) of the SAFRC at all doses, and R_0_, R_1_ (i.e., RT = R_2_) of the BPP at high doses showed significant differences compared to controls (*p* ≤ 0.05).

Figure 5 showed the results of Petri dish 4. Only the GP_1_ and the GP_2_ were also measured. Compared with the control, R_0_ (i.e., RT = R_1_) of the GP_1_ at all doses and R_0_ (i.e., RT = R_1_) of the GP_2_ at high doses had more than 15% differences.

Figure 6 showed the results of Petri dish 5. Compared with the control, there was no generations with more than 15% differences in the GP_1_ (i.e., RT = R_0_) and the GP_2_ (i.e., RT = R_0_). The results of the SOD activity was complicated: there were significant differences in R_0_ and R_1_, while the R_3_ or the R_4_ at a dose also showed significant differences (*p* ≤ 0.05). After induction, we finalized the RT of the SOD activity as R_2_. For the SAFRC, the significant differences were observed in R_0_, R_1_ (i.e., RT = R_2_) at the highest dose and R_0_ (i.e., RT = R_1_) at other doses compared with the control (*p* ≤ 0.05). For the BPP, there is no significant differences in all generations (i.e., RT = R_0_) compared with the control (*p* ≤ 0.05).

Figure 7 showed the results of Petri dish 6. Only the GP_1_ and the GP_2_ were also measured. There were no more than 15% different generations compared with the respective control, thus, the RT of both the GP_1_ and the GP_2_ were R_0_. The results of the analysis in the Region 1–6 from heavy ion beam irradiation were also listed in Table 1.

### DES processing trials (Fig. 8)

Because the plantlets with normal germination were selected for chemical mutagens treatment, there was no R_0_ determination of the GP_1_ and the GP_2_ (see "Methods" for details). Compared with the control, there were no generations (i.e., RT = R_1_) with more than 15% difference in the GP_1_ and the GP_2_. For the SOD activity, the SAFRC and the BPP, compared with the respective controls, there were only significant differences in R_0_ (i.e., RT = R_1_) at high doses, whereas other treated generations (i.e., RT = R_0_) had no significant differences (*p* ≤ 0.05) at low doses. The analysis results were also listed in Table 1.

### MH processing trials (Fig. 9)

MH was completely consistent with DES processing results. The analysis results were also listed in Table 1.

### RT analysis at seed germination stage

Using the RT of GP_1_ or GP_2_ may characterize this performance (Table 1 as shown). The GP_1_ may reflect the uniformity of seed germination, whereas the GP_2_ may reflect the survival ability of the seeds and the intensity of the action of the various mutagenic factors to the seeds. Table 1 indicated that the variation trend of the RTs of both the GP_1_ and the GP_2_ was consistent, compared with the untreated control group. For the irradiation in the gamma-rays and the Plateau regions and the Tail regions along carbon ion beam pathway at low doses, the RTs of the GP_1_ and the GP_2_ were R_0_, whereas the RTs of the GP_1_ and the GP_2_ of the treatments from two chemical mutagens and the Peak regions along carbon ion beam pathway were R_1_.

### RT analysis at seedling stage

Using the RT of SOD activity or SAFRC may characterize their performances (Table 1 as shown). The SOD activity may reflect the cell stress facing to the mutagenic factors, while the SAFRC cannot only reflect the stress of the cells, but also reveals the indirect effects of various mutagenic factors, the results are shown in Table 1. It showed that the RT of SODA was R_1_ after the treatments of low dose gamma rays, the Tail region along carbon ion beam pathway and two chemical mutagens, compared with untreated control group; the RT of the Peak region along carbon ion beam pathway was R_3_; and the RT of the other treatment group was R_2_. The treatment of gamma-ray, low-dose Tail region along carbon ion beam pathways and two chemical mutagens were performed, the RT of the SAFRC was R_1_; the RT of the SAFRC the Plateau region and the Peak region along carbon ion beam pathway were R_3_ and R_4_, respectively.

### RT analysis at plant maturation stage

The performance of biomass per plant was characterized by the RT (Table 1 as shown). The BPP cannot only reflect the growth of *Astragalus sinicus* L. plants, but also reflect the synthesis and accumulation of plant cell metabolites. Table 1 revealed that the RT of the BPP of gamma-ray, the Plateau region and the Tail region along carbon ion beam pathway as well as chemical mutagens was mainly R_0_ or R_1_, compared with the untreated control group, whereas the RT of the BPP of the Peak region at high-doses along carbon ion beam pathway was R_2_.

## Discussion

In general, the RT is significantly different not only among different mutagenic factors and but also among different biological effects. The RT of gamma rays and chemical mutagens is relatively short. After planting a generation, except for mutants, the population affected by them can basically restore homeostasis. Whereas the results of the action of the heavy ion beams are the most complex, and the RT distribution of its biological effects is from R_0_ to R_4_, which may be related to the existence of a variety of physicochemical mechanisms such as energy deposition, mass deposition, charge effects, and momentum transfer along different tracks of the heavy ion beams^9,19^, especially in the treatment of the Peak region, some biological effects requires a longer time to restore the population homeostasis. The longer RT after irradiation by heavy ion beams may reflect the accumulation of DNA mutations and chromosome rearrangements. Generally, heavy ion beams are more mutagenic than gamma rays, diethyl sulfate, or maleyl hydrazine^23–25^.

According to the RT of different biological effects, it has positive application value in at least three aspects. First, during the breeding of mutagenesis mutants, the different strategy can be adopted: generally speaking, because of the longer stable time on the population, the biological trait with long Relaxation Time needs more generations to choose and accumulate useful individual plants. Second, in the study of stress and adaptability of biological individuals, it can be distinguished based on the length of Relaxation Time: the biological effects of short RT are stress traits, while the biological effects of long RT are adaptive traits. The third is that, in the biological study of phylogeny, because the organisms have experienced the action of natural environment factors for a long time, seeking and using some primitive or starting population as the control, the concept of the RT can still be introduced. By comparing the RT difference of specific biological effects among populations, the way of some population evolution can be speculated. Especially in the era of bioomics popularity, from the gene expression and metabolic pathway changes to metabolic network connectivity, we can quickly obtain the comparison results of related indicators such as nucleotide sequences between different species or individuals, which can be easily characterized by their RTs.

Not only is the biology a complex system^16–18^, but its mutation is also a complex processing. as a result, there will be a rebalancing procedure among differently biological macromolecules (such as DNA, RNA, proteins and chromosome), different organelles (such as mitochondrion) and different metabolic pathways (such as antioxidant systems regulated by ROS). Thus, fully revealing the RTs of those life processes and inherited characters will be our important research objectives.

## Methods

### Gamma irradiation

In 2015, the seeds of *Astragalus sinicus* L. harvested in 2014 were arranged around the irradiation chamber near the ^60^Co source (the sources active was 221,754 Ci) at the Tianjin Irradiation Center. The radius of dose rates of 20, 10 and 2 Gy/min from the center of the ^60^Co source bar was 0.87 m, 1.46 m and 4.19 m at the sites of 30 cm high, respectively. the seeds were placed in these three sites and irradiated for 10 min to obtain the irradiated materials with absorption doses of 200,100 and 20 Gy, respectively.

### Irradiation by carbon ion beams

**Figure 10:**
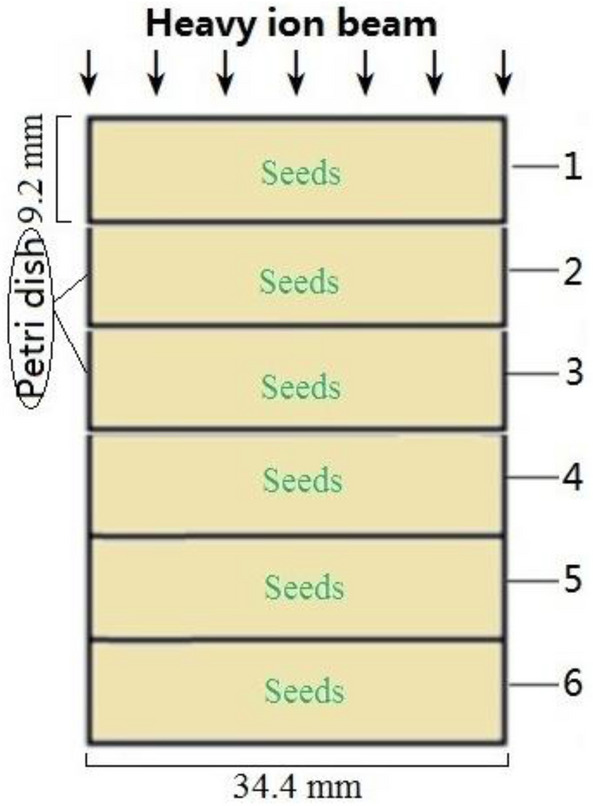
Schematic diagram of the seeds irradiated by heavy ion beams in Petri dishes.

Because 827 heavy ions of 80 meV/u carbon ions is equivalent to the dose of 1 Gy, an average dose rate was 0.5 Gy/min when the beam intensity of 400 ~ 500 ions/s used at this test. The doses of 20, 100 and 200 Gy needed 40, 200 and 400 s of irradiation time, respectively. In 2015, the seeds of *Astragalus sinicus* L. harvested in 2014 were filled with a Nunc plastic Petri dishes (34.4 mm diameter, 9.2 mm thick), and the six Petri dishes were stacked to be used in irradiation test with 80 meV/u carbon ion beams on the TR_4_ terminal of the Lanzhou Heavy Ion Accelerator (Fig. 10). According to the theoretical calculation (Lise^++^9.9: https://lise.nscl.msu.edu/lise.html) and the measurement of CR-39 heavy ion track detectors^23,24^, when 87.5 meV/u of carbon ion beams was used to test, the Bragg peak of the energy loss appears near 2.2 cm depth in water and between 2.8 and 3.4 cm depth in Arabidopsis seeds along the tracks (Wang et al.^25^). While 80 meV/u of carbon ion beams was used to this test, the Bragg peak of the energy loss appears between 2.2 and 3.2 cm depth (not spread-out Bragg peak) in *Astragalus sinicus* L. seeds along the tracks (about at the Petri dish 3). After irradiation, the whole installation was divided into six Petri dishes, which were classified as Petri dish 1–6 from close to far away according to the contact of carbon ion beams: Petri dish 1 of the Plateau region 1, Petri dish 2 of the Plateau region 2, Petri dish 3 of the Peak region, Petri dish 4 of the Peak-tail connect region, Petri dish 5 of the tail region 1 and Petri dish 6 of the tail region 2. The seeds of each Petri dish were applied to subsequent experiments.

### Treatment of chemical mutagens

**Figure 11:**
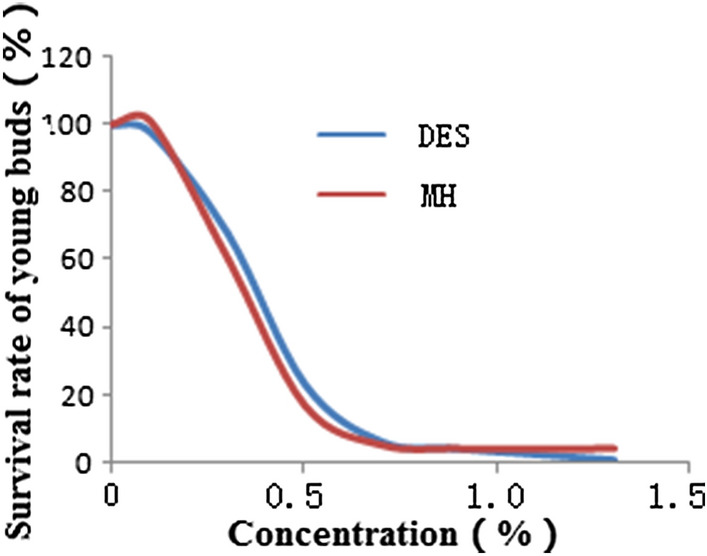
Survival curves of seedlings of *Astragalus sinicus* L.treated with chemical mutagens. DES, diethyl sulfate. MH, maleyl hydrazine.

The whole seedlings of *Astragalus sinicus* L. (at 1–2 leaf stage) were treated with different concentrations (0–1.3%) of diethyl sulfate (DES) and maleyl hydrazine (MH) solutions at room temperature for 24 h, respectively. After washing with the sterilization water, their survival curves were plotted by statistics on survival ratios (as shown in Fig. 11). According to Fig. 11, the working concentrations of DES and MH of 0, 0.1%, 0.3% and 0.5% were used to test the seedlings. In 2015, the seeds of *Astragalus sinicus* L. harvested in 2014 were germinated. After about 10 days (at 1–2 leaf stage), the above working concentrations of DES and MH solution were sprayed to the whole seedlings of R_0_ generation for 24 h, respectively. After washing with the sterilization water, we sampled and carried out cultivation successions according to different treatments for subsequent trials. Just because of this kind of treatment, the germination potential and germination percentage of R_0_ generation were not counted in this paper.

### Germination test

A Petri dish (a diameter of 150 mm and depth of 25 mm) with seeds was used for the germination test at room temperature and natural light. The germination potential (GP_1_) was counted by the number of *Astragalus sinicus* L. germination within 7 days, and the germination percentage (GP_2_) was counted by the number of all germination seeds about 20 days before transplanting. The chemical mutagen treatment group performed germination potential and germination percentage statistics from the R_1_ generation seeds (i.e., the seeds harvested by the R_0_-generation plants), while the other treatment groups performed germination potential and germination percentage statistics from the R_0_ generation (the starting seeds harvested in 2014).

### Determination of SOD activity and Superoxide anion free radical content

At the later stage of the germination test (about 20 days, including seedlings with chemical inducer treatment), the whole plant of 20 seedlings was randomly selected from each dish, and the crude extract of seedlings was extracted according to Xiao HS, et al.^26^ and stored in a refrigerator at —20℃ for the determination of SOD activity (SODA) and Superoxide anion free radical content (SAFRC), which the determination process was carried out according to the method from Xiao HS, et al.^26^ and Simova-Stoilova LK, et al.^27^.

### Field trials and determination of biomass per plant

All seedlings were transplanted into the field after about 20 days of indoor germination (including chemical mutagen-treated seedlings). three repeated, random block design with a plot of 12 m^2^, and the plant spacing was 30 cm (North–South) × 20 cm (East–West) to ensure that each individual plant can grow normally and was rarely affected by other plants. Up to maturity, the single plant was harvested, full dried indoor and performed test seeds, respectively. And finally the average biomass per plant (BPP) will be calculated according to the plot block.

### Data statistics

A total of about 100 seeds of *Astragalus sinicus* L. were combined and added up after germination from two Petri dishes, and then the germination potential and germination percentage were counted. Compared with the control materials of the same generation without any treatment, when there was more than 15% mean of a treatment group, this treatment was acted as a significant difference (Indicated by #). SOD activity and Superoxide anion free radical content were took the OD value of survival seedling of 2 germination dishes to determine their effect, respectively, which every effect was calculated by its average value (Mean) and standard deviation (SD). The Mean and SD of Biomass per plant were calculated according to 3 plot blocks. Compared with control materials without any treatment at the same generation, when the statistics variance of SOD activity, Superoxide anion free radical content or Biomass per plant, was evaluated by *p* ≤ 0.05, the biological effect was regarded as a significant difference (Expressed by an asterisk *).

## Supplementary information

Supplementary information 1.

Supplementary information 2.

Supplementary information 3.
